# Chemotherapy Reverses Anti-PD-1 Resistance in One Patient With Advanced Non-small Lung Cell Cancer

**DOI:** 10.3389/fonc.2020.00507

**Published:** 2020-04-21

**Authors:** Lingdi Zhao, Baozhen Ma, Yonghao Yang, Tiepeng Li, Lu Han, Quanli Gao

**Affiliations:** Department of Immunotherapy, Henan Cancer Hospital, Affiliated Cancer Hospital of Zhengzhou University, Zhengzhou, China

**Keywords:** immunotherapy, chemotherapy, lung cancer, pembrolizumab, resistance

## Abstract

**Background:** Programmed cell death protein 1(PD-1) blockade has become a standard second-line treatment option for patients with advanced non-small cell lung cancer (NSCLC) without a driver gene mutation. Previous clinical studies showed that the objective response rate (ORR) of PD-1 blockade as second-line treatment for patients with NSCLC was ~20%, and the median progression-free survival (PFS) was ~4 months, with most patients eventually developing a resistance to PD-1 blockade. Although the ORR to chemotherapy after PD-1 blockade resistance was relatively high, the survival time of patients could not be significantly prolonged. Clinical oncologists are unclear about which treatment regimen should be selected after PD-1 blockade failure. Here, we report about a patient with advanced NSCLC and initial PD-1 blockade resistance who was observed to have a rapid partial response (PR) following one dose of chemotherapy and subsequent PD-1 blockade treatment.

**Case presentation:** A 70-year-old woman with a history of left lower lobe lung surgery in March 2018 (pathological stage T1N2M0, EGFR wild-type) presented to our hospital. After six cycles of adjuvant chemotherapy, multiple nodules in both the lungs developed, and were suspected to be metastatic lesions. After another 2 months, the nodules in both the lungs enlarged. From November 2018 to March 2019, the patient received six cycles of pembrolizumab, and computed tomography (CT) confirmed a progressive disease status. She was then managed with 260 mg/m^2^ albumin paclitaxel once every 3 weeks. Subsequently, chemotherapy was discontinued after one cycle owing to grade three neuromuscular toxicity. Follow-up CT revealed a stable disease in May 2019. She then received another six cycles of pembrolizumab, which resulted in a PR.

**Conclusion:** Chemotherapy may play a role in reversing PD-1 blockade resistance. If failure of PD-1 blockade occurs at first, re-administration of PD-1 blockade may be implemented if first followed by several cycles of chemotherapy. Because there are few reports on the use of chemotherapy to reverse PD-1 resistance, it is necessary to conduct clinical studies with larger patient cohorts to investigate whether chemotherapy can reverse PD-1 blockade resistance.

## Background

The emergence of immune checkpoint inhibitors represented by anti-PD-1/PD-L1 antibodies has changed the treatment of many kinds of malignant tumors and indicates the advent of the era of immunotherapy. Currently, anti-PD-1 antibodies have been approved for second-line or later line treatment of multiple advanced malignancies. Specifically, pembrolizumab has been approved for patients with advanced NSCLC without driver gene mutations, if the PD-L1 expression is over 50% (1–5)5. As second-line therapy for advanced NSCLC, the ORR of PD-1 blockade was ~20%, indicating that most patients did not benefit from PD-1 blockade (1, 2). Combination therapy that includes PD-1 blockade has also been used for treating a variety of solid tumors. The combination therapy which includes PD-1 blockade is called the 2.0 era of immunotherapy. Even in patients that have shown to benefit from PD-1 blockade, some develop resistance to PD-1 blockade eventually. For most patients with PD-1 blockade resistance, there are no effective treatment options at the present time. Some studies have reported outcomes associated with PD-1 blockade combined with chemotherapy, antiangiogenic therapy, and radiotherapy after failure of PD-1 blockade alone; these studies also report outcomes on those who were switched to chemotherapy and antiangiogenic therapy alone (6–9). However, there are no reports on the efficacy of re-administration of PD-1 blockade after single dose of chemotherapy in patients with PD-1 blockade resistance. Here, we report about a patient with advanced NSCLC who initially demonstrated PD-1 blockade resistance and then showed partial response following re-administration PD-1 blockade after one cycle of chemotherapy.

## Case Presentation

**Figure 1:**
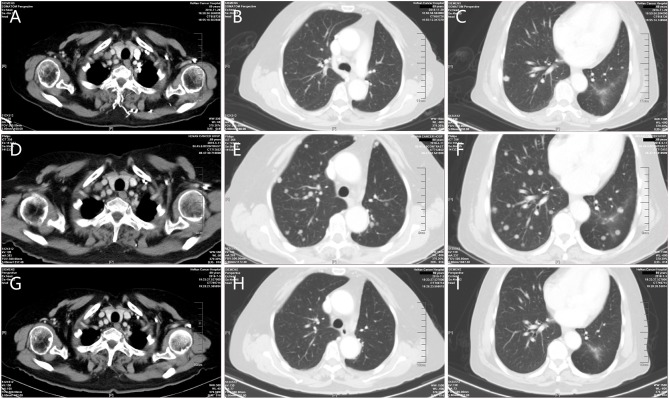
Representative computed tomography (CT) obtained throughout the clinical course. **(A–C)** CT during initial therapy (prior to the first time of pembrolizumab administration). **(D–F)** Progressive disease while receiving pembrolizumab therapy. **(G–I)** Significant reduction in tumor burden following the fourth dose of pembrolizumab after re-administration.

A 70-year old woman was diagnosed with lung cancer in March 2018 and therefore subsequently underwent surgery on March 28, 2018. Postoperative pathology showed adenocarcinoma, consisting of acinar, and papillary structures with a tumor associated vascular thrombus; 10 of the 14 lymph nodes examined were positive for cancer cells. The postoperative pathological stage was T1N2M0. From May to September 2018 she received six cycles of adjuvant chemotherapy with pemetrexed plus lobaplatin. Multiple nodules were noted on follow-up CT in September 2018. Two months following this, the nodules enlarged according to CT (Figures 1A–C). She received six cycles of pembrolizumab at a dose of 2 mg/kg every 3 weeks. The efficacy evaluation was immune progressive disease in February 2019 and confirmed progressive disease in April 2019 (Figures 1D–F). She received albumin paclitaxel at a dose of 260 mg/m^2^ once every 3 weeks starting in April 2019. During this time grade three neuromuscular toxicity and grade two neutropenia were observed. CT showed stable disease in May 2019 and following this she refused to receive additional chemotherapy. Pembrolizumab was re-administered at the same dose from May to September 2019, and follow-up CT showed that the lung nodules had reduced in size and PR was observed (**Figures 1G-I**). Pembrolizumab was tolerated well, and grade one fatigue and grade one hypothyroidism occurred during the treatment.

## Discussion And Conclusions

PD-1 blockade has become the standard second-line treatment for patients with advanced NSCLC without driver gene mutations; however, the ORR was found to be ~20% and the mean PFS was ~4 months (1, 2). Most of the patients will develop primary resistance or secondary resistance and there is no effective treatment option for these patients who show resistance. It was reported that the ORR to platinum-containing doublets in patients with advanced NSCLC after failure of PD-1 blockade was over 50%, and the ORR to single-agent chemotherapy was ~40% (10, 11). For patients with gastrointestinal cancers, the disease control rate was high with chemotherapy or target therapy after failure of PD-1 blockade (12). To date, there are no reports on effective PD-1 blockade re-administration after one dose of single-agent chemotherapy. Here we report a patent who received re-administration of PD-1 blockade and showed PR after one dose of single-agent chemotherapy after failure of an initial attempt with PD-1 blockadealone. The patient's response demonstrated a possible new treatment approach for patients after PD-1 blockade resistance.

Chemotherapeutic agents could induce immunogenic and non-immunogenic death of tumor cells, and at the same time could play immunomodulatory functions by reducing regulatory T cells and myeloid-derived suppressor cells by remodeling the immune microenvironment, and by working synergistically with chemotherapy as shown in some clinical studies (13, 14). After chemotherapy, T cell proliferation decreased and PD-L1 up-regulated, which were in accordance with the concept of tumor escape in tumor microenvironment (15, 16). Although it was shown that 5-Fluorouracil could selectively deplete myeloid-derived suppressor cells *in vivo*, and oxaliplatin could trigger an ICD, Dosset et al. demonstrated that 5-Fluorouracil plus oxaliplatin drove complete tumor cure in mice when combined to anti-PD-1 treatment, while each monotherapy failed (15). One *in-vitro* study showed immunogenic tumor antigen expression was increased 4-fold in human ovarian cancer cells pretreated with paclitaxel (17). In patients with resectable breast cancer, the response to neoadjuvant paclitaxel correlated with an increase in tumor infiltrating lymphocytes before surgery (18). Moreover, the application of albumin paclitaxel eliminated the need for glucocorticoid pretreatment and therefore eliminated the adverse effects of glucocorticoids on lymphocytes. Antiangiogenic agents could reshape the tumor microenvironment and make it toward for the immunologically supported tumor microenvironment (19). In theory, chemotherapy or anti-angiogenesis plus PD-1 blockade could potentially produce a synergistic effect.

The mechanisms of PD-1 blockade resistance mainly involve effector cells and the tumor microenvironment. For example, resistance is related to insufficient T lymphocytes that infiltrate the tumor microenvironment, poor specificity of cytotoxic T cells with an inability to recognize tumor antigens effectively, poor response of cytotoxic T cells to PD-1 signaling and T lymphocyte suppression independent of PD-1/PD-L1 signals. The tumor microenvironment is associated with resistance due to poor immunogenicity of tumor cells, poor sensitivity of tumor cells to cytotoxic T cells, and poor presenting function of antigen presenting cells. Therefore, strategies could be designed for different resistance mechanisms to restore the sensitivity of tumor cells to T cells and reverse PD-1 blockade resistance (20). In clinical practice, PD-1 blockade combined with chemotherapy, antiangiogenic therapy, radiotherapy after failure of PD-1 blockade, or a switch to chemotherapy and antiangiogenic therapy are often provided to patients with PD-1 blockade resistance. We have also witnessed good results with PD-1 blockade plus chemotherapy or anti-angiogenesis after PD-1 blockade resistance (not published).

The treatment mode presented in this case is different from those mentioned in current clinical studies and those currently used in clinical practice. The tumor was not sensitive to PD-1 blockade, but after only one dose of albumin paclitaxel, the tumor became sensitive to PD-1 blockade and partial response was observed. It was reported that PR could be achieved after 6 months with PD-1 blockade (21). Honestly, PR achieved in this patient could not be excluded completely by PD-1 blockade monotherapy. In this patient, PR was achieved about 10 months after the first dose of pembrolizumab. During treatment, the first PD occurred in Feb 2019 and confirmed PD in April 2019. At the same time, the patient felt mild chest pain and shortness of breath after exercise, which could be attributed from the enlargement of the tumors in lung and pleura. Therefore, the probability of true progression for this patient was very high. This indicated the complexity of treatment in patients with PD-1 blockade resistance. We still have a long way to go in fully understanding the best treatment for these patients.

This case report indicates that one cycle of single-agent chemotherapy may be effective in reversing PD-1 blockade resistance in patients with advanced NSCLC. It should be noted that the PD-1 blockade was ineffective at first and became effective after one cycle of single-agent chemotherapy. This phenomenon supports the idea that chemotherapy may change the tumor microenvironment and make it more sensitive to immunotherapy. The mechanism underlying this is still unclear and needs to be further explored.

## Data Availability Statement

The data used to support the findings of this study are included within the article.

## Ethics Statement

The studies involving human participants were reviewed and approved by Henan Cancer Hospital Medical Ethics Committee. The patients/participants provided their written informed consent to participate in this study. This article has passed the ethical review for paper publication.

## Author Contributions

LZ was responsible for the conception and drafting the manuscript. BM, YY, TL, and LH were responsible for the date collection and figures. QG was responsible for conception and edition of the manuscript. All authors read and approved the final manuscript.

## Conflict of Interest

The authors declare that the research was conducted in the absence of any commercial or financial relationships that could be construed as a potential conflict of interest.

## Abbreviations

PD-1: programmed cell death protein 1
NSCLC: non-small cell lung cancer
ORR: objective response rate
PFS: median progression-free survival
PR: partial response
CT: computed tomography.
